# A novel gene signature for prognosis prediction and chemotherapy response in patients with pancreatic cancer

**DOI:** 10.18632/aging.202922

**Published:** 2021-04-26

**Authors:** Hongcao Lin, Chonghui Hu, Shangyou Zheng, Xiang Zhang, Rufu Chen, Quanbo Zhou

**Affiliations:** 1 Guangdong Provincial Key Laboratory of Malignant Tumor Epigenetics and Gene Regulation, Sun Yat-Sen Memorial Hospital, Sun Yat-Sen University, Guangzhou, Guangdong Province, China; 2 Department of Pancreatobiliary Surgery, Sun Yat-Sen Memorial Hospital, Sun Yat-Sen University, Guangzhou, Guangdong Province, China; 3 Department of General Surgery, Guangdong Provincial People's Hospital, Guangdong Academy of Medical Sciences, Guangzhou, Guangdong Province, China

**Keywords:** pancreatic cancer, chemoresistance, tumor microenvironment, overall survival, nomogram

## Abstract

Pancreatic cancer is a lethal disease. Chemoresistance is one of the characteristics of pancreatic cancer and leads to a poor prognosis. This study built an effective predictive model for personalized treatment and explored the molecular mechanism of chemoresistance. A four-gene signature, including serine peptidase inhibitor Kazal type 1 (*SPINK1*), anoctamin 1 (*ANO1*), desmoglein 3 (*DSG3*) and GTPase, IMAP family member 1 (*GIMAP1*) was identified and associated with prognosis and chemoresistance in the training group. An internal testing dataset and the external dataset, GSE57495, were used for validation and showed a good performance of the gene signature. The high-risk group was enriched with multiple oncological pathways related to immunosuppression and was correlated with epidermal growth factor receptor (EGFR) expression, a target molecule of Erlotinib. In conclusion, this study identified a four-gene signature and established two nomograms for predicting prognosis and chemotherapy responses in patients with pancreatic cancer. The clinical value of the nomogram was evaluated by decision curve analysis (DCA). It showed that these may be helpful for clinical treatment decision-making and the discovery of the potential molecular mechanism and therapy targets for pancreatic cancer.

## INTRODUCTION

Pancreatic cancer is one of the highly malignant tumors associated with a 5-year survival rate as low as 9%, and ranks fourth as a common cause of cancer-associated death in the USA [1]. However, it is estimated to become the second common cause of cancer-associated death before 2030 [2]. Surgical resection is considered the only potentially curative treatment, but less than 10% of patients are resectable using the standard resection, and the 5-year survival rate of these early stage patients is only 24.6% [3]. Systemic chemotherapy, radiotherapy, and targeted molecular therapy are also treatment choices for postoperative patients or patients with unresectable tumors [4]. Recurrence, early metastasis, and resistance to chemoradiotherapy are important characteristics of pancreatic cancer [5]. Therefore, patients with pancreatic cancer should have individualized systemic treatment to prolong survival time and improve quality of life; thus, it is essential to identify an effective predictive prognosis model and biomarkers for guiding individualized systemic treatment. Clinical characteristics including the American Joint Committee on Cancer (AJCC) stage and pathological type are commonly used as indicators for prognosis and treatment evaluation [6]. With the development of gene chips and high-throughput sequencing technology, prediction tools based on prognosis-related genes have been developed with better accuracy, and may be beneficial to elucidate the molecular mechanism of pancreatic cancer occurrence and progression.

One of the important factors for poor prognosis in pancreatic cancer is chemoresistance, including extrinsic or intrinsic resistance [7, 8]. Intrinsic resistance is driven by multiple mechanisms that are not clearly understood. For instance, Farrell JJ et al. has found that human equilibrative nucleoside transporter 1 (hENT1), a transport protein that transports gemcitabine and other nucleoside analogs into cellular compartments, has variable expression in patients with pancreatic cancer with different responses to chemotherapy [8, 9]. Ju HQ et al. has found that gemcitabine resistance in pancreatic cancer plays a role through redox modulation [10]. Extrinsic resistance results from changes in the stromal microenvironment, including dense fibrotic tumor stroma and immunosuppression [11–13]. At present, gemcitabine or S-1, a fluoropyrimidine derivative is commonly used as a first-line chemotherapy drug, while (m)FOLFIRINOX and gemcitabine plus nanoparticle albumin-bound paclitaxel are other choices for patients who can tolerate regimens [4]. With the continuous development of molecular biology technology, the molecular mechanisms of chemoresistance have been studied, and clinical trials of combination therapies, targeted molecular drugs, and immunotherapy are ongoing [14]. However, these studies have not contributed much to improving the survival of patients with pancreatic cancer. Erlotinib, a target inhibitor of the epidermal growth factor receptor (EGFR) tyrosine kinase, is currently recommended as a combination treatment for patients with skin rash, and is beneficial to the overall survival of patients with pancreatic cancer [4, 15]; but, immunotherapy has no efficacy in patients with pancreatic cancer due to the immunosuppressive tumor microenvironment [13].

Many gene signatures have been reported to be related to prognosis [16, 17]. However, they are seldom associated with the chemotherapy response. In this study, a four-gene prognostic signature related to the chemotherapy response was constructed based on the different responses after chemotherapy in The Cancer Genome Atlas (TCGA) and the Genotype-Tissue Expression (GTEx) project datasets training group, and validated in both an internal testing group and an external Gene Expression Omnibus (GEO) dataset. Moreover, the potential molecular mechanisms were explored through gene set enrichment analysis (GSEA) and tumor immunity relevance analysis. Two clinical nomogram models were also constructed to predict prognosis and the chemotherapy response and might serve as a reference for chemotherapy in patients with pancreatic cancer.

## RESULTS

### Identification of resistance-related differentially expressed genes (RRDEGs)

**Figure 1:**
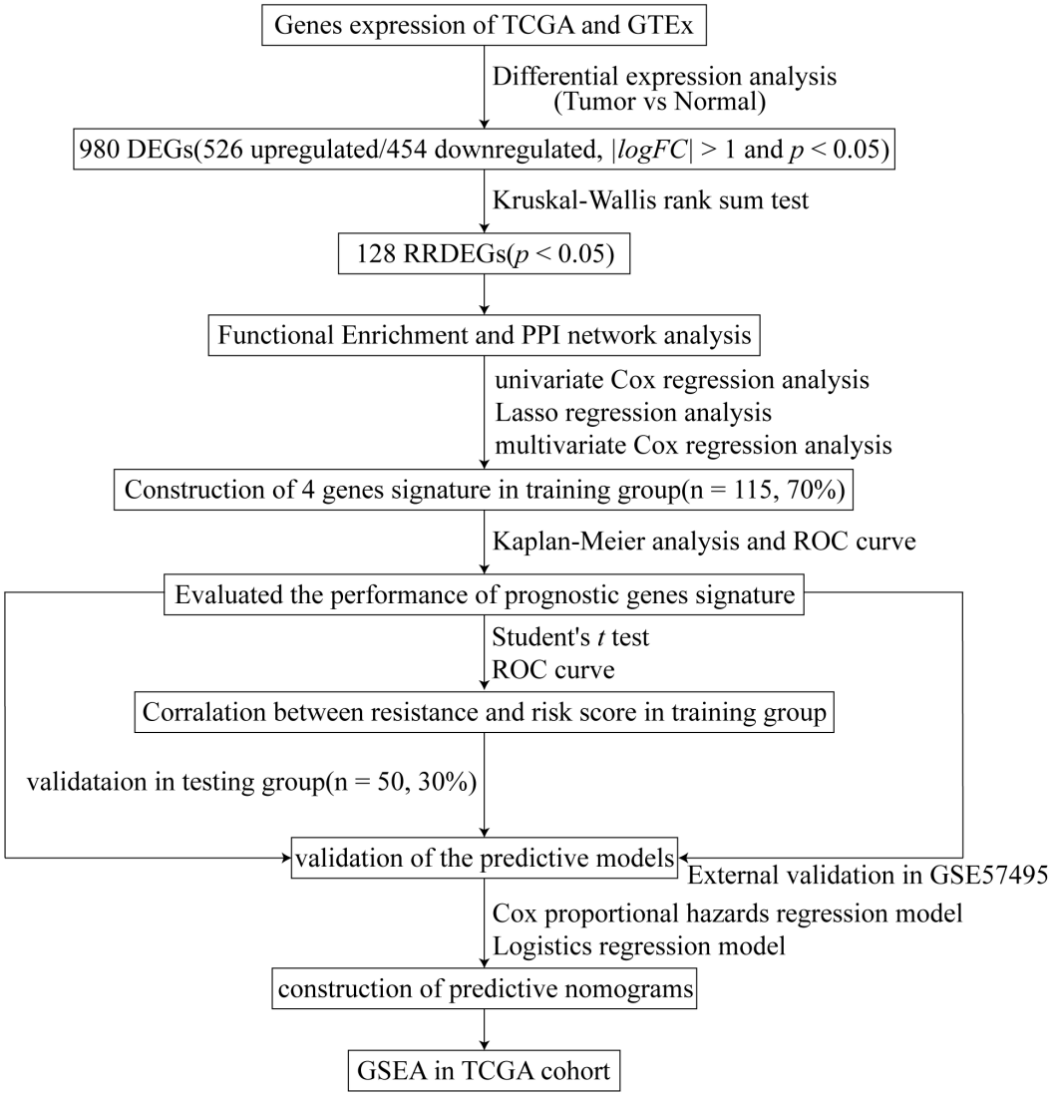
Study flowchart.

**Figure 2:**
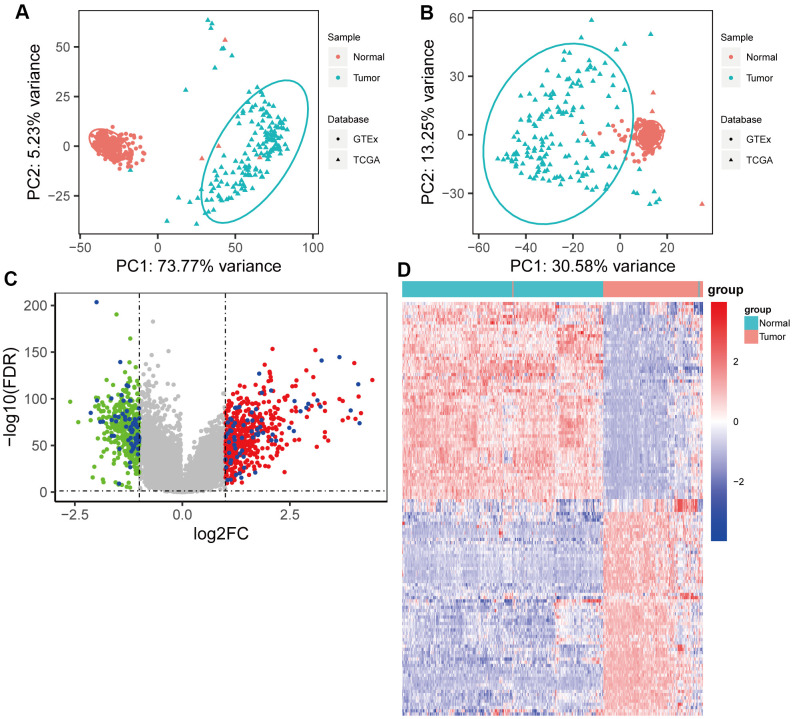
**Identification of resistance-related differentially expressed genes (RRDEGs) in pancreatic cancer.** (**A**) Principle component analysis (PCA) plot of the data merged from The Cancer Genome Atlas (TCGA) and Genotype-Tissue Expression (GTEx) datasets before removing the batch effect. (**B**) PCA plot of the data merged from TCGA and GTEx datasets after removing the batch effect. (**C**) Volcano plot of DEGs between tumor tissues and normal tissues. The red and green points are DEGs, and RRDEGs are plotted with blue points. The lines were drawn where the absolute value of log2FC is equal to 1 and FDR is equal to 0.05. (**D**) Heatmap showing the expression of RRDEGs.

Figure 1 depicts a flowchart of the study. The clinical characteristics of patients with pancreatic cancer in TCGA dataset are shown in Supplementary Table 1. TCGA and GTEx datasets with 165 pancreatic cancer samples and 332 normal samples were used to conduct differential expression analysis between tumor samples and normal samples after removing the batch effect (Figure 2A, 2B); 980 differential expression genes (DEGs) (526 upregulated and 454 downregulated genes) were identified (|*log2FC*| > 1 and *p* < 0.05). As chemoresistance plays a significant role in the progression of pancreatic cancer, the expression of 71 patients with different responses after chemotherapy in the TCGA database was analyzed using a Kruskal-Wallis rank sum test. A total of 128 RRDEGs were identified (*p* < 0.05, Figure 2C, 2D and Supplementary Table 2).

### GO and KEGG enrichment analysis of RRDEGs

**Figure 3:**
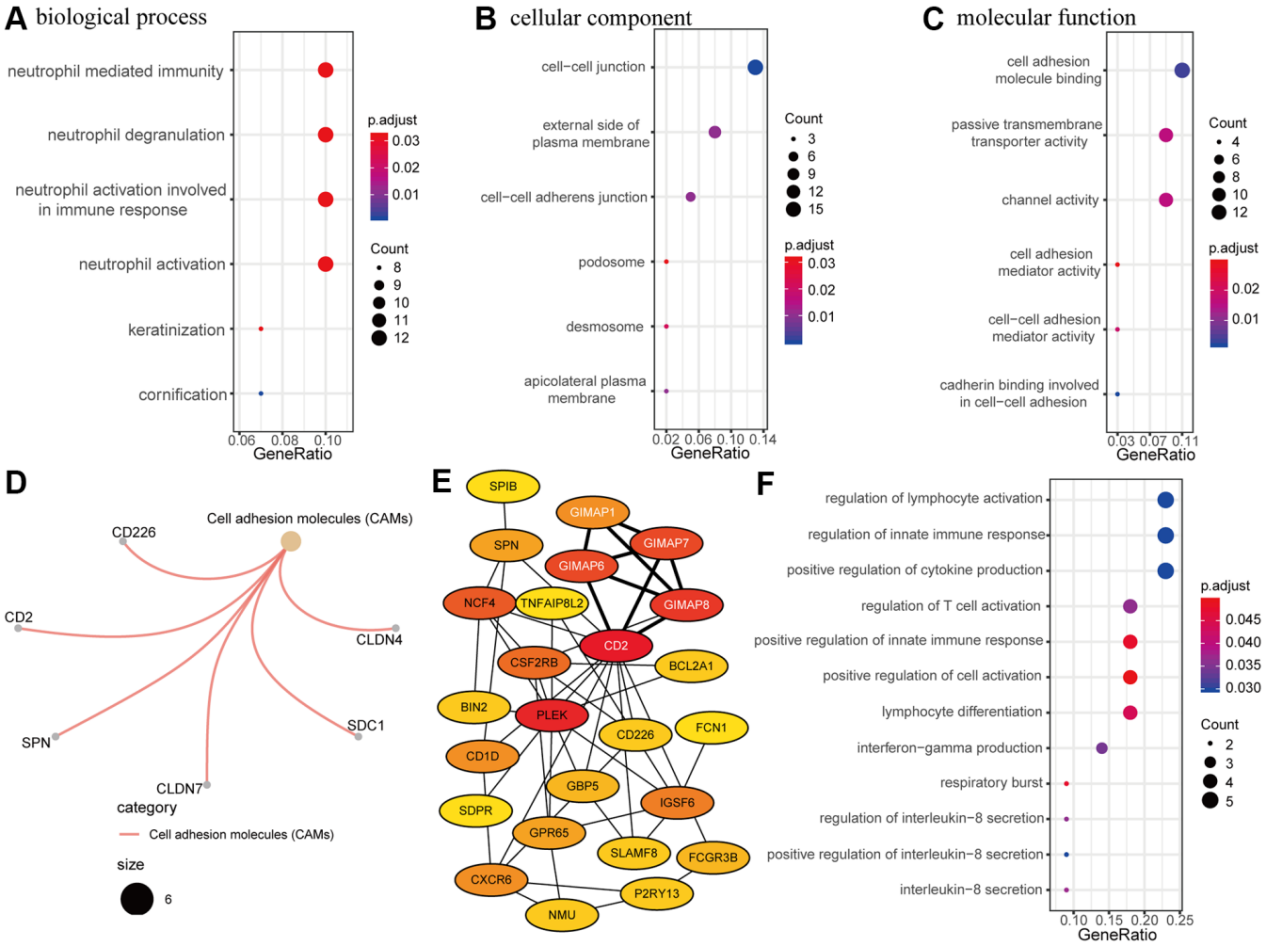
**Functional enrichment and protein-protein interaction (PPI) network analysis.** (**A**–**C**) Gene ontology (GO) enrichment analysis of resistance-related differentially expressed genes (RRDEGs). (**A**) Biological process. (**B**) Cellular component. (**C**) Molecular function. (**D**) Kyoto Encyclopedia of Genes and Genomes (KEGG) analysis of RRDEGs. (**E**) Visualization of the top 25 hub genes in the PPI network. A clustering module with a score of 4.5 is marked with white font. (**F**) GO enrichment analysis of hub genes in the biological process.

To explore RRDEGs function, RRDEGs were subjected to GO and KEGG enrichment analysis (Supplementary Table 3). RRDEGs were enriched in biological processes related to neutrophils, such as neutrophil degranulation and activation, which suggested that innate immunity plays an important role in tumor progression and chemoresistance (Figure 3A). GO enrichment analysis in cellular component and molecular function indicated that the RRDEGs were related to cell adhesion, which was consistent with the highly aggressive nature of pancreatic cancer and extrinsic resistance mechanisms (Figure 3B, 3C). Furthermore, KEGG pathway analysis also revealed that RRDEGs were related to cell adhesion (Figure 3D).

A PPI network of RRDEGs was created to identify protein interactions; the top candidate hub included 25 genes that played a significant role in this network (Figure 3E). Module analysis identified a meaningful clustering module with score 4.5 in the PPI network, which was marked with white font on the network (Figure 3E). Functional enrichment analysis of biological processes for these hub genes was associated with the immune response (Figure 3F), including the innate immune response, cytokine production, and lymphocyte activation. Thus, PPI network analysis showed that RRDEGs promoted pancreatic cancer progression and chemoresistance through immune regulation.

### Construction of a four-gene signature associated with chemoresistance

**Figure 4:**
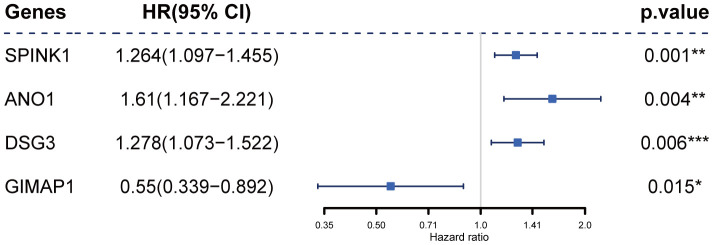
Construction of the prognostic four-gene signature using a Cox regression model.

Next, univariate Cox regression analysis was performed to identify potential prognostic RRDEGs in the training group; 51 RRDEGs were identified that were significantly associated with overall survival (Supplementary Table 4). Lasso-penalized Cox analysis was then used to eliminate model over-fitting and showed that the expression of 10 RRDEGs was correlated with overall survival in patients with pancreatic cancer (Supplementary Figure 1A, 1B and Supplementary Table 5). Subsequently, a prognostic signature composed of four genes, including anoctamin 1 (*ANO1*), desmoglein 3 (*DSG3*), serine peptidase inhibitor Kazal type 1 (*SPINK1*) and GTPase, IMAP family member 1 (*GIMAP1*), was identified using stepwise multivariate Cox regression analysis (Figure 4). The downregulated gene *GIMAP1*, with a hazard ratio (*HR*) < 1, was regarded as a tumor suppressor, while the upregulated genes *ANO1*, *DSG3*, and *SPINK1*, with a *HR* > 1, were considered oncogenes. The risk score was calculated using the following formula:

$$\begin{array}{l} \text{Risk}\;\text{score}=(0.47599\times \text{Expression value}\;\text{of}\;\text{ANO}1)\\ \;\;\;\;\;\;\;\;\;\;\;\;\;\;\;+(0.24553\times \;\text{Expression value of}\;\text{DSG}3)\\ \;\;\;\;\;\;\;\;\;\;\;\;\;\;\;+(0.23389\times \;\text{Expression value of}\;SPINK1)\\ \;\;\;\;\;\;\;\;\;\;\;\;\;\;\;+[(-0.59799)\times \;\text{Expression}\;\text{value}\;\text{of}\;\text{GIMAP}1]. \end{array}$$

**Figure 5:**
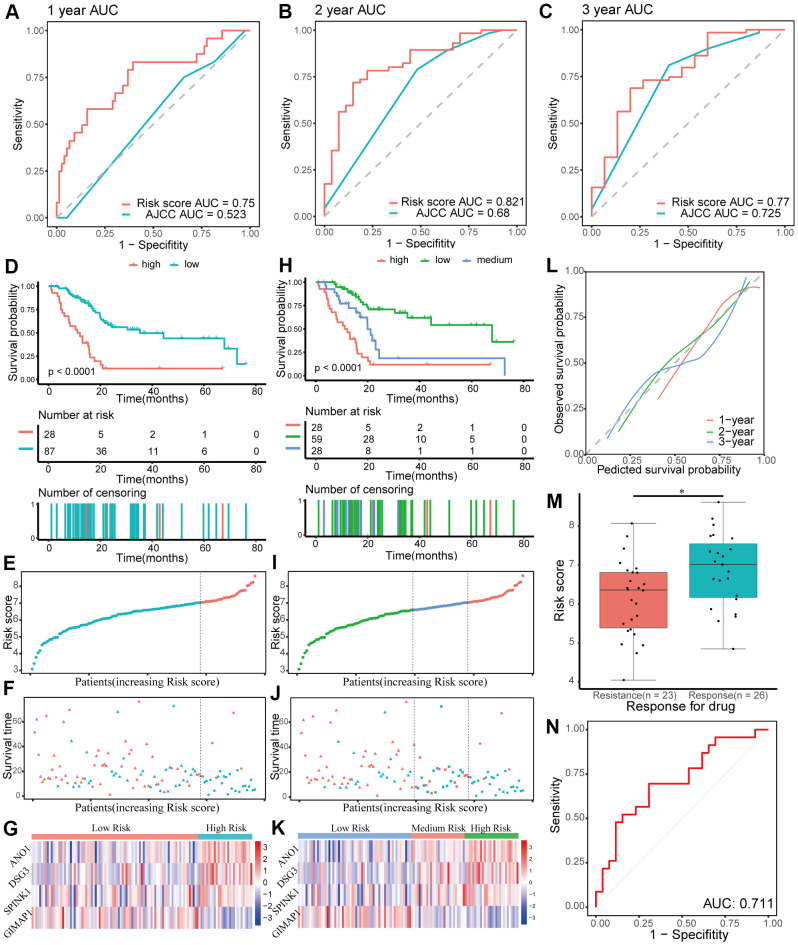
**Evaluating the performance of the prognostic gene signature in the training group.** (**A**–**C**) Time-dependent receiver operating characteristic (ROC) curves for 1-, 2-, and 3-year overall survival predictions of the gene signature. American Joint Committee on Cancer (AJCC) stage is the control. (**D**–**G**) Kaplan-Meier survival curves of the four-gene signature and distribution of patient survival and risk score in different groups when patients are divided into two groups. (**D**) Kaplan-Meier survival curves. (**E**) Distribution of risk score. (**F**) Distribution of survival time. Circle shape stands for high-risk group while triangle shape for low-risk group. Red stands for survival and green stands for dead. (**G**) Heatmap of the expression of the four genes. (**H**–**K**) Kaplan-Meier survival curves of the four-gene signature and distribution of patient survival and risk score in different groups when patients are divided into three groups. (**H**) Kaplan-Meier survival curves. (**I**) Distribution of risk score. (**J**) Distribution of survival time. (**K**) Heatmap of the expression of the four genes. (**L**) Calibration plot for validation of the gene signature. (**M**) Distribution of risk score in different responses to drug in the training group. (**N**) The ROC curve for the response to drug prediction of risk score in the training group is shown.

The patients in the training group were divided into two (cutoff value = 7.03) or three (cutoff values = 6.59 and 7.03, respectively) groups based on the optimal cutoff values generated by the X-tile software. Time-dependent ROC and C-index analyses were used to evaluate the prognostic value of the four-gene signature compared to the AJCC stage. The area under the curves (AUCs) for 1-, 2-, and 3-year overall survival predicted by the risk scores were 0.750 (95% CI: 0.631–0.869), 0.821 (95% CI: 0.725–0.916), and 0.770 (95% CI: 0.629–0.911), respectively (Figure 5A–5C). As a control, the AUCs for 1-, 2-, and 3-year overall survival predicted by the AJCC stage were 0.523 (95% CI: 0.419–0.628), 0.680 (95% CI: 0.569–0.791), and 0.725 (95% CI: 0.589–0.861), respectively. The C-index of the gene signature was 0.724 (95% CI: 0.650–0.798), while the C-index of the AJCC stage was 0.573(95% CI: 0.504–0.643). Kaplan-Meier survival curve analysis showed that patients with lower risk scores had a significantly more favorable prognosis (Figure 5D–5K). The calibration curve for the gene signature demonstrated a satisfactory fit in the training group (Figure 5L).

Chemoresistance is an important factor for poor progression of pancreatic cancer. In this study, the prognostic risk score based on patients with different responses after chemotherapy was closely related to chemoresistance in the training group (*p* < 0.05, Figure 5M). Moreover, the AUC for chemoresistance predicted by the risk score was 0.711(95% CI: 0.563–0.858, Figure 5N). In general, these data showed that the four-gene signature performed well in predicting prognosis and chemoresistance of pancreatic cancer.

### Internal and external validation of the four-gene signature

**Figure 6:**
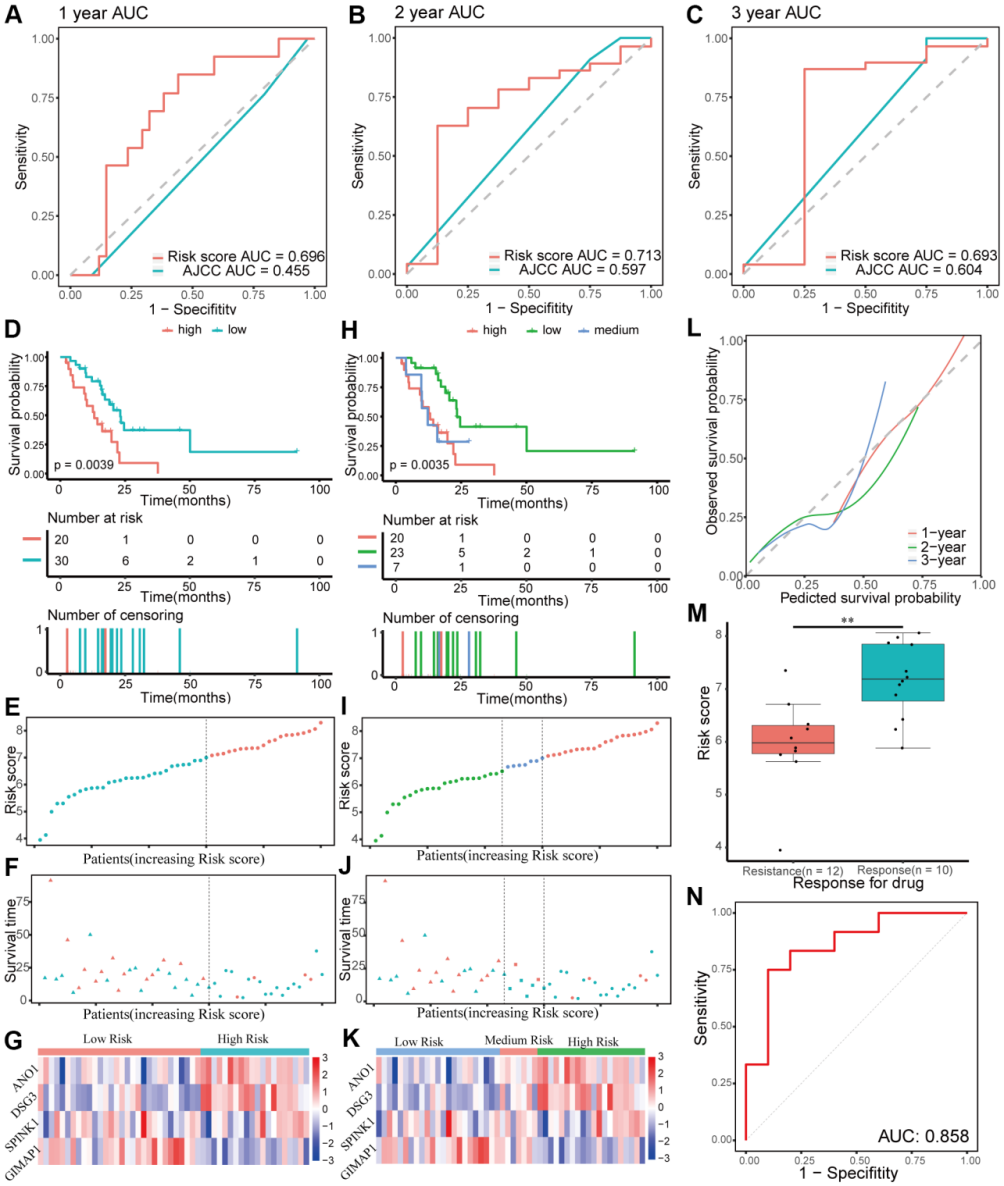
**Validation of the prognostic gene signature in the testing group.** (**A**–**C**) Time-dependent receiver operating characteristic (ROC) curves for 1-, 2-, and 3-year overall survival predictions of the gene signature in the testing group. American Joint Committee on Cancer (AJCC) stage is the control. (**D**–**G**) Kaplan-Meier survival curves of the four-gene signature and distribution of patient survival and risk score in different groups when patients in the testing group are divided into two groups. (**D**) Kaplan-Meier survival curves. (**E**) Distribution of risk score. (**F**) Distribution of survival time. Circle shape stands for high-risk group while triangle shape for low-risk group. Red stands for survival and green stands for dead. (**G**) Heatmap of the expression of the four genes. (**H**–**K**) Kaplan-Meier survival curves of the four-gene signature and distribution of patient survival and risk score in different groups when patients in the testing group are divided into three groups. (**H**) Kaplan-Meier survival curves. (**I**) Distribution of risk score. (**J**) Distribution of survival time. (**K**) Heatmap of the expression of the four genes. (**L**) Calibration plot for validation of the gene signature in the testing group. (**M**) Distribution of risk score in different responses to drug in the testing group. (**N**) The ROC curve for the drug reaction prediction of risk score in the testing group is shown.

The performance of the gene signature was validated in the internal testing group and external GEO dataset, GSE57495. Risk scores were calculated according to the same formula for each patient. In the testing group, patients were divided into two or three groups using the same cutoff values used in the training group. Due to sequencing using different platforms, patients in the GEO dataset were divided into two (cutoff value = 6.71) or three (cutoff values = 6.24 and 6.90, respectively) groups according to the optimal cutoff values. Time-dependent ROC and C-index analyses were utilized to elevate the prognostic predictive value of the four-gene signature compared with the AJCC stage. In the testing group, the AUCs for 1-, 2-, and 3-year overall survival predicted by the risk score were 0.696, 0.713, and 0.693, respectively (Figure 6A–6C). The AUCs for 1-, 2-, and 3-year overall survival predicted by the AJCC stage were 0.455, 0.597, and 0.604, respectively. The C-index of the gene signature was 0.639 (95% CI: 0.540–0.737), while the C-index of the AJCC stage was 0.522 (95% CI: 0.431–0.614). In the external GEO dataset, the AUCs for 1-, 2-, and 3-year overall survival predicted by the risk score were 0.615, 0.653, and 0.674, respectively (Supplementary Figure 2A–2C), while the AUCs for the AJCC stage were 0.575, 0.682, and 0.584, respectively. The C-index of the gene signature for the GEO dataset was 0.603 (95% CI: 0.528–0.677), while the C-index of the AJCC stage was 0.594 (95% CI: 0.509–0.680). Kaplan-Meier survival curve analysis showed significant differences for overall survival between the different risk score groups both in the testing group and the external GEO dataset (*p* < 0.05, Figure 6D–6K and Supplementary Figure 2D–2K). Furthermore, the calibration curve for the gene signature revealed that the predicted overall survival was approximately consistent with the actual overall survival (Figure 6L and Supplementary Figure 2L). However, when the predicted 3-year overall survival in the testing group was higher than 50%, the signature might underestimate the overall survival in patients with pancreatic cancer. In the testing group, the correlation between response to chemotherapy and risk score was also explored and showed that patients with chemoresistance had a higher risk score (*p* < 0.01, Figure 6M). Moreover, the AUC for chemoresistance predicted by the risk score was 0.858 (95% CI: 0.695–1, Figure 6N). The internal and external validation indicated that the four-gene signature performed well in predicting overall survival and chemoresistance in patients with pancreatic cancer.

### Building predictive nomograms

Patients with complete clinical information from TCGA dataset were included in the analysis. The clinical characteristics included gender, age, AJCC TNM stage, tumor dimension, tumor site, pathologic grade, chemotherapy, neoadjuvants, radiation, molecular therapy, residual tumor after surgery, tobacco smoking history, alcohol use history, history of chronic pancreatitis, and diabetes. Prognostic factors of overall survival for pancreatic cancer were identified using univariate and stepwise multivariate Cox regression analyses while chemoresistance predictive factors for pancreatic cancer were identified using univariate and stepwise multivariate logistics regression analyses.

**Figure 7:**
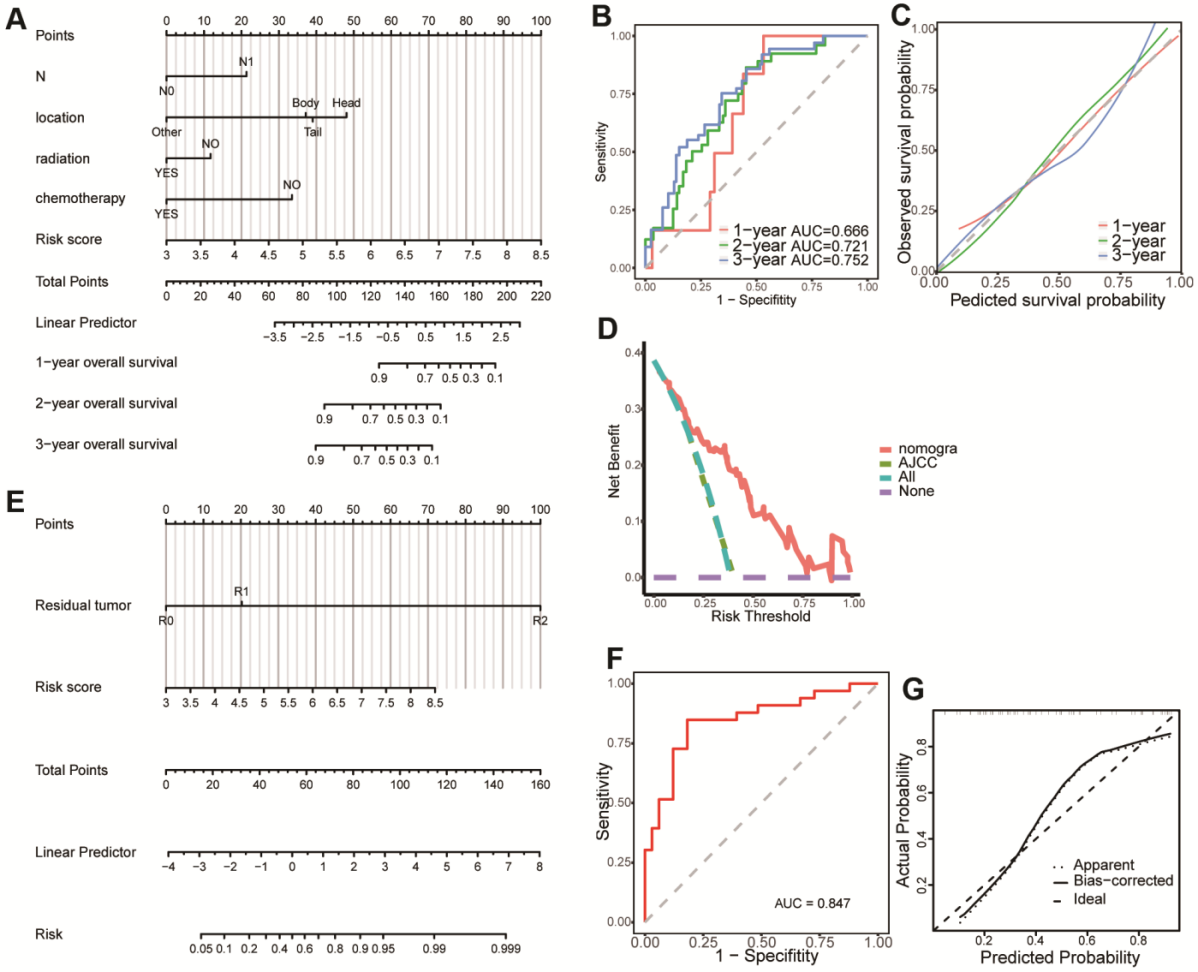
**Creation of a predictive nomogram in The Cancer Genome Atlas (TCGA) dataset.** (**A**) A prognostic nomogram predicting 1-, 2-, and 3-year overall survival of patients with pancreatic cancer. (**B**) Time-dependent receiver operating characteristic (ROC) curves for 1-, 2-, and 3-year overall survival predictions of the nomogram. (**C**) Calibration plot for the validation of the prognostic nomogram. (**D**) The nomogram predicting OS were compared against AJCC stage by DCA. (**E**) A predictive nomogram predicting drug reaction in pancreatic cancer. (**F**) ROC curve of the nomogram for the prediction of chemotherapy resistance. (**G**) Calibration plot for the validation of the chemotherapy resistance predictive nomogram.

Univariate Cox regression analysis revealed that risk score (*p* < 0.001), AJCC stage (*p* < 0.05), T stage (*p* < 0.05), N stage (*p* < 0.05), tumor dimension (*p* < 0.05), tumor site (*p* < 0.01), pathologic grade (*p* < 0.05), chemotherapy (*p* < 0.05), radiation (*p* < 0.05), and molecular therapy (*p* < 0.01) were corrected with overall survival of patients with pancreatic cancer. Stepwise multivariate Cox regression analysis indicated that the risk score (*p* < 0.001), N stage (*p* < 0.01, N1 vs N0), and chemotherapy (*p* < 0.001) were significantly correlated with overall survival of patients with pancreatic cancer. A prognostic nomogram predicting 1-, 2-, and 3-year overall survival based on the stepwise multivariate Cox regression model was developed (Figure 7A). The AUC of the 1-, 2-, and 3-year overall survival predictions for the nomogram was 0.666, 0.721, and 0.752, respectively (Figure 7B). The C-index of the nomogram was 0.791 (95% CI: 0.732–0.849). The calibration plot showed that the nomogram was effective at predicting 1-, 2-, and 3- year overall survival in patients with pancreatic cancer (Figure 7C). In the DCA, the results showed that the nomogram indicated a better net benefit than was achieved with the AJCC stage for predicting OS (Figure 7D).

Univariate logistics regression analysis revealed that risk score (*p* < 0.001), residual tumor after surgery (*p* < 0.05, R1 vs R0), and N stage (*p* < 0.05) were corrected with response after chemotherapy. Additionally, stepwise logistics regression analysis showed that the risk score (*p* < 0.01) and residual tumor after surgery (*p* < 0.05, R1 vs R0) were independent risk factors of disease progression after chemotherapy. A predictive nomogram predicting the probability of response after chemotherapy was built based on the logistics regression model (Figure 7E). The AUC of the disease progression probability predictions for the nomogram was 0.847 (Figure 7F) while the C-index of the nomogram was 0.847. The calibration plot showed good agreement between predictions and observations (Figure 7G).

### GSEA and tumor immunity relevance of the gene signature

**Figure 8:**
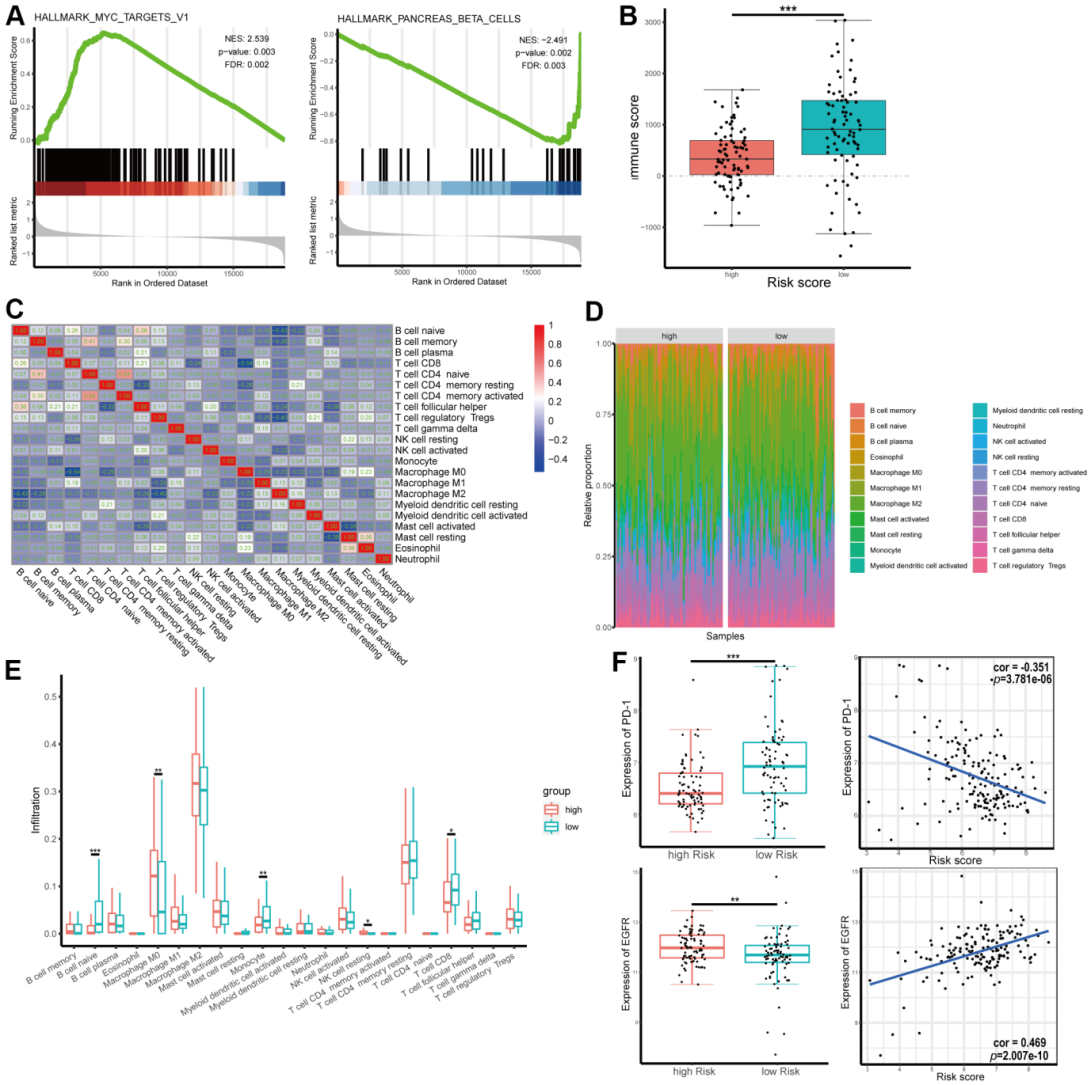
**Gene set enrichment analysis (GSEA) and tumor immunity relevance of the gene signature.** (**A**) GSEA for hallmark gene sets. Upregulated and downregulated enriched pathways with top normalized enrichment scores are shown. (**B**) Distribution of immune score in high and low risk groups in The Cancer Genome Atlas (TCGA) dataset. (**C**) Correlation of the infiltration proportion between different immunocytes. (**D**, **E**) Infiltration levels of different immunocytes in high and low risk groups. (**F**) Correlation between expression of programmed cell death protein 1 (PD-1) or epidermal growth factor receptor (EGFR) and risk score.

To elucidate the molecular mechanisms of the four-gene signature, 165 patients in TCGA dataset were divided into two groups according to the median of the risk score and GSEA was used to compare the high and low risk groups. The results revealed the malignant characteristics of cancer and immunity relevance and included cholangiocarcinoma, breast cancer, pancreas beta cell, *MYC*, keratinization, and mutation of *P53* and *KRAS* (Figure 8A and Supplementary Figure 3A–3C). Results of the GSEA are shown in Supplementary Table 6.

As shown above, RRDEGs might be related to tumor immunity. To further investigate the tumor immunity relevance of the gene signature, immune scores of TCGA-PAAD samples were downloaded from ESTIMATE, a database evaluating infiltrating immune cells in tumor tissues (https://bioinformatics.mdanderson.org/estimate/), and infiltration information of different immunocytes in TCGA-PAAD samples was downloaded from TIMER (http://timer.cistrome.org/). The immune score was significantly lower in the high-risk group, which revealed that the four-gene signature might play an important role in tumor immune escape (Figure 8B). Immune cell proportions were weakly to moderately correlated in patients with pancreatic cancer (Figure 8C). Infiltration levels of different immunocytes, including naïve B cells, macrophage M0s, monocytes and CD8+ T cells, were significantly different between different risk groups (Figure 8D, 8E). To evaluate the correlation between risk score and immunotherapy, correlation analysis between the expression of the immune checkpoint programmed cell death protein 1 (PD-1) and risk score was performed; the results showed that risk score was negatively correlated with the expression level of PD-1 (correlation coefficient = -0.351, Figure 8F). However, there was not significance difference between signature and the expression level of PD-L1 (Supplementary Figure 4A). The expression of EGFR, a target molecule of Erlotinib, was positively correlated with risk score (correlation coefficient = 0.469, Figure 8F), which suggested that these risk scores might be used as a reference for Erlotinib-targeted drug therapy. To further explore the role and the clinical relevance of genes, we analyzed the expression level of mRNA from dataset. The mRNA expression of *SPINK1*, *DSG3*, *ANO1* were significantly increased in PAAD tumor tissue while *GIMAP1* were significantly decreased compared with normal tissues (Supplementary Figure 4B–4E).

## DISCUSSION

Pancreatic cancer is a lethal disease where the 5-year survival rate is only 9% [1]. However, current treatments, including surgery, chemotherapy, radiation therapy, targeted molecular therapy, and immunotherapy have only led to modest improvements in overall survival. Accurate prognostic models provide a reference for personalized treatments strategies, which are expected to improve patient prognosis. The AJCC stage and other clinical features are commonly used as prognostic markers and are one of the reference indicators for clinical treatment strategies. Additionally, molecular prognostic markers provide good supplementation to the AJCC stage for improving accuracy of prognosis predictions [6]. Moreover, chemoresistance plays a significant role in the progress of pancreatic cancer, leads to poor prognosis, and may have effects through a complex molecular regulatory network [5, 7, 8, 12, 18, 19]. By analyzing the related molecules, these molecules can be used as prognostic molecular markers and help in the understanding of the mechanism of chemoresistance, which may provide new therapeutic targets. Nomograms that combine several prognostic risk factors, including molecular markers and clinical characteristics, are widely used to accurately calculate and visualize the probabilities of clinical events, and contribute to clinical decisions in personalized treatment strategies [20–22].

In this study, a four-gene prognostic signature that included *SPINK1*, *ANO1*, *DSG3*, and *GIMAP1* in patients with pancreatic cancer were identified. The gene signature was related to the chemotherapy response based on different responses in patients after chemotherapy in training group, and was validated in both an internal testing group and an external GEO dataset. The gene signature was closely correlated with prognosis, chemoresistance, and target molecular therapy of patients with pancreatic cancer. To explore the potential mechanisms of these genes in pancreatic cancer progression and chemoresistance, the patients were divided into high and low risk groups based on risk score and GSEA was performed. When using oncogenic gene sets, a correlation was revealed with *KRAS* and *P53*, common genes with mutation in pancreatic cancer; it has been reported that *KRAS* and *P53* are associated with chemoresistance [23, 24]. Utilizing hallmark gene sets, *MYC*-related pathways and other pathways are enriched, which is consistent with previous studies where *MYC* has been reported to play a role in cell growth, proliferation, and tumorigenesis in pancreatic cancer [25, 26]. Subsequently, it was found that patients with a high-risk score had a lower immune score, which suggested that the high-risk group had a tumor immunosuppressive microenvironment. Furthermore, the relationship between risk score and tumor immunotherapy was explored and the results show that risk score is negatively correlated with the expression level of PD-1, which may be due to the immunosuppressive microenvironment and is consistent with the modest curative effect of anti-PD-1 drugs in pancreatic cancer [27]. Although there is no benefit for immunotherapy in pancreatic cancer [13], new regimens of immunotherapy may be one of the choices in the future.

*SPINK1*, a trypsin inhibitor, is secreted into pancreatic juice by pancreatic acinar cells. It is encoded by *SPINK1*, located in chromosomal region 5q32. Mutation of this gene is closely associated with idiopathic chronic pancreatitis [28], a risk factor of pancreatic cancer. However, the relationship between *SPINK1* and the development of pancreatic cancer is controversial [29, 30]. Chen F et al. has found that overexpression of *SPINK1* promotes pancreatic cancer aggressiveness, particularly chemoresistance, through the epithelial-endothelial transition mediated by EGFR downstream signaling [31]. However, a meta-analysis shows that *SPINK1* has no correlation with the progression of pancreatic cancer [30]. Overexpression of *SPINK1* has also been discovered in multiple tumors, including colon, lung, breast, and prostate, and is associated with tumor progression and poor prognosis [32, 33]. *ANO1*, a calcium-activated chloride channel, is a biomarker for poor prognosis in pancreatic cancer. Overexpression of *ANO1* promotes pancreatic cancer cell migration via the ligand-dependent EGFR signaling pathway [34, 35]. *ANO1* is also upregulated in multiple tumor tissues, including head and neck squamous cell carcinoma (HNSCC), and prostate and breast cancer [36, 37]. It is reported that *ANO1* interacts with EGFR and affects EGFR-targeted therapy in HNSCC and breast cancer [38, 39]. However, additional experiments are required to elucidate whether this occurs in pancreatic cancer. *DSG3*, encoded by *DSG3*, located in chromosomal region 18q12.1, is a member of the desmoglein family and the cadherin cell adhesion molecule superfamily of proteins that establish links between adjacent cells [40]. *DSG3* has been identified as an autoantigen in the skin disease pemphigus vulgaris [41] and is also upregulated in several cancers, including squamous cell carcinoma, pancreatic ductal adenocarcinoma, and head and neck cancer [40, 42, 43]. In pancreatic cancer, it has been identified as a negative prognosis biomarker [40]. GIMAP1, a guanosine triphosphatase (GTPase) that is immunity-associated, is involved in Th cell differentiation and development of mature B and T lymphocytes [44]. GIMAP1 has been commonly reported to be related to autoimmune diseases, such as Bechet’s disease [45] and type I diabetes [46]. Moreover, GIMAP1 is reported to be downregulated in lymphomas and regulates the B and T lymphocyte cell cycle [47]. However, there are no reports on its effect in pancreatic cancer.

There are limitations to this study. First, the model was conducted and validated in TCGA and GEO datasets and some clinical information was limited, especially the information on chemotherapy. Therefore, it is necessary to validate the model in a larger number of samples with complete clinical information. Second, the potential molecular mechanisms of progression and chemoresistance in pancreatic cancer that were identified in this study need to be verified by further experimental studies.

This study identified a four-gene signature that predicted prognosis and chemoresistance in patients with pancreatic cancer. In addition, two nomograms were established in pancreatic cancer. The gene signature was closely correlated with prognosis, chemoresistance, and target molecular therapy of patients with pancreatic cancer and might be beneficial to the discovery of potential mechanisms of progression and chemoresistance. Two predictive nomograms were created to be used as references for clinical treatment decisions.

## MATERIALS AND METHODS

### Gene expression data and clinical data

The gene expression profiles (HTSeq-Counts) of patients with pancreatic cancer (TCGA-PAAD) and their associated drug information were downloaded from TCGA using the R package “TCGAbiolinks,” while the other clinical information was obtained from the cBioPortal database (https://www.cbioportal.org/). Samples meeting the following criteria were excluded: (1) a metastatic tumor; (2) without pathological information; (3) with a pathological diagnosis of colloid (mucinous non-cystic) carcinoma or undifferentiated carcinoma; and (4) follow-up time less than 30 days. Meanwhile, eight cases without corresponding gene expression data were eliminated. Therefore, 165 cases, 165 tumor samples and 4 normal samples, were included in this study. Gene expression profiles (gene read counts) from normal pancreatic tissues (n = 328) of healthy individuals were downloaded from the GTEx project (https://www.gtexportal.org/). Then, TCGA and GTEx gene expression data were combined for further analysis. The gene expression microarray dataset, GSE57495, associated with follow-up information, was downloaded from GEO (https://www.ncbi.nlm.nih.gov/geo/) for the validation of the prognostic model.

### Differentially expressed gene (DEG) identification and analysis

Gene expression data were normalized and a variance stabilizing transformation using the R package “DESeq2” was applied. To reduce the influence of a batch effect, a batch variate giving rise to a different database was designed as a covariant, and the batch effect was further removed using the removeBatchEffect () function in the R package “limma.” DEGs between tumor samples and normal samples were identified using “limma.” |*log_2_FC*| > 1 and *p* < 0.05 were set as the cutoffs for DEGs. Then, RRDEGs were identified using a Kruskal-Wallis rank sum test between patients with different responses after chemotherapy, including “Clinical Progressive Disease”, “Partial Response”, “Stable Disease” and “Complete Response”. *p* < 0.05 was considered statistically different. Furthermore, gene ontology (GO) enrichment and Kyoto Encyclopedia of Genes and Genomes (KEGG) pathway analyses were performed for RRDEGs using the R package “clusterProfiler.” A protein–protein interaction (PPI) network was conducted using the search tool for the retrieval of interacting genes/proteins (STRING) database (https://www.string-db.org/) and was visualized using Cytoscape v. 3.7.2 (http://www.cytoscape.org/).

### Construction of a risk prognostic signature

A total of 165 tumor samples were randomly divided into two groups, a training group (*n* = 115, 70%) and a testing group (*n* = 50, 30%). There were 49 samples with chemotherapy information in the training group, while 22 samples had chemotherapy information in the testing group. A risk prognostic model was constructed based on the data from the training group and validated in the testing group and the GSE57495 dataset. First, a univariate Cox proportional hazards regression model was performed to identify potential survival-related RRDEGs for subsequent analysis. Lasso-penalized Cox regression analysis was performed to reduce over-fitting based on the “glmnet” package. Next, a stepwise multivariate Cox proportional hazards regression model was used to construct a risk prognostic signature, and the risk score was calculated through the summation of the gene expression multiplied by the regression coefficients from the multivariate Cox proportional hazards regression model. Patients with different responses after chemotherapy were divided into two groups. Patients with a response from “Clinical Progressive Disease” were assigned to one group and the others were assigned to another group. A student’s *t*-test was performed to evaluate the correlation between risk score and chemoresistance. The receiver operating characteristic (ROC) curve was performed to evaluate the predictive power of the gene signature for chemoresistance. Patients were separated into two or three groups based on an optimal cutoff value risk score using X-tile software (V3.6.1). Kaplan-Meier analysis, ROC curves, Harrell’s concordance indexes (C-index), and calibration plots were conducted to evaluate the performance of the prognostic gene signature. The AJCC stage was used as a control. The GSE57495 dataset, with follow-up information, was set as an external validation dataset and the same formula was used to calculate the risk score.

### Construction of nomograms and DCA

To identify independent risk factors of prognosis, univariate and multivariate Cox regression analyses were performed, and clinical characteristics included age, gender, AJCC stage, T, N, M, dimension, location, grade, histopathology, neoadjuvant, radiation, molecular therapy, residual tumor, smoking, drinking, pancreatitis, diabetes, chemotherapy, and risk score. *p* < 0.05 was considered statistically different. All independent risk factors were included in the construction of prognostic nomograms using a stepwise Cox regression model in TCGA PAAD dataset. A univariate and multivariate binary logistics regression model was performed to identify chemoresistance risk factors, and all were included in the construction of a predictive nomogram using a stepwise method. The ROC curve, C-index, calibration plots, and DCA were conducted to evaluate the performance of the nomograms.

### GSEA

GSEA was used to explore the potential molecular mechanism of a prognostic gene signature. All patients in TCGA dataset were divided into two groups according to the median of the risk score. The R package “limma” was used to acquire the DEGs between high and low risk groups, and GSEA was performed using the R package “clusterProfiler” [48], based on the Molecular Signatures Database v. 7.0. Hallmark gene sets included C2 (curated gene sets), C5 (GO gene sets) and C6 (oncogenic signatures). *p* < 0.05 was regarded as statistically different.

### Data of tumor immunity relevance in pancreatic cancer

The data on tumor purity and the presence of infiltrating stromal/immune cells in TCGA PAAD sample tumor tissue were download from the Estimation of STromal and Immune cells in MAlignant Tumor tissues using Expression data (ESTIMATE) [49] (https://bioinformatics.mdanderson.org/estimate/). The abundance of different immunocyte immune infiltrates' in TCGA PAAD samples was downloaded from TIMER (http://timer.cistrome.org/) [50] and the CIBERSORT method was used to acquire the results [51].

### Statistical analysis

Statistical analysis was performed in the programming language R (v3.6.2, https://www.r-project.org/). Pearson's chi-squared test was used to analyze categorical variables. A Wilcoxon signed rank test or Student’s *t*-test was used to compare two groups of continuous variables. A Kruskal-Wallis rank sum test was used to analyze multiple groups of continuous variables. Lasso regression analysis, and univariate and multivariate Cox regression analyses were performed for survival analysis. Univariate and multivariate binary logistics regression analysis were performed for regression analysis of binary category variables. *p* < 0.05 was regarded as statistically different.

## Supplementary Material

Supplementary Figures

Supplementary Table 1

Supplementary Table 2

Supplementary Table 3

Supplementary Table 4

Supplementary Table 5

Supplementary Table 6
