# Supplementary material for: A novel gene signature for prognosis prediction and chemotherapy response in patients with pancreatic cancer
Source: Aging (Albany NY). 2021 Apr 26;13(9):12493–513. doi: 10.18632/aging.202922 (PMC8148498; doi:10.18632/aging.202922)
Supplement: Supplementary Figures [file aging-13-202922-s001.pdf]

## SUPPLEMENTARY FIGURES

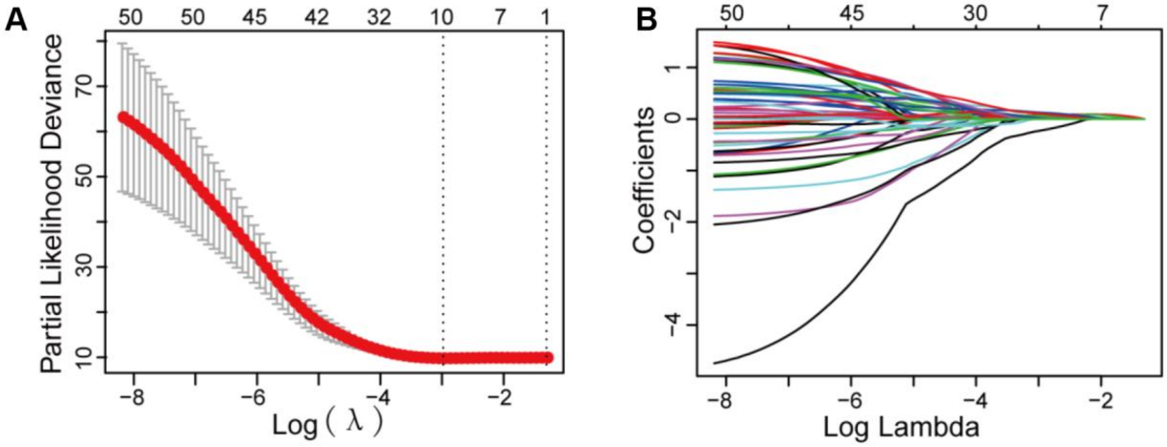


**Supplementary Figure 1. Lasso analysis of the prognostic RRDEGs in pancreatic cancer.** (**A**) Selection of tuning parameter (λ) in LASSO model. (**B**) LASSO coefficient profiles of all genes.

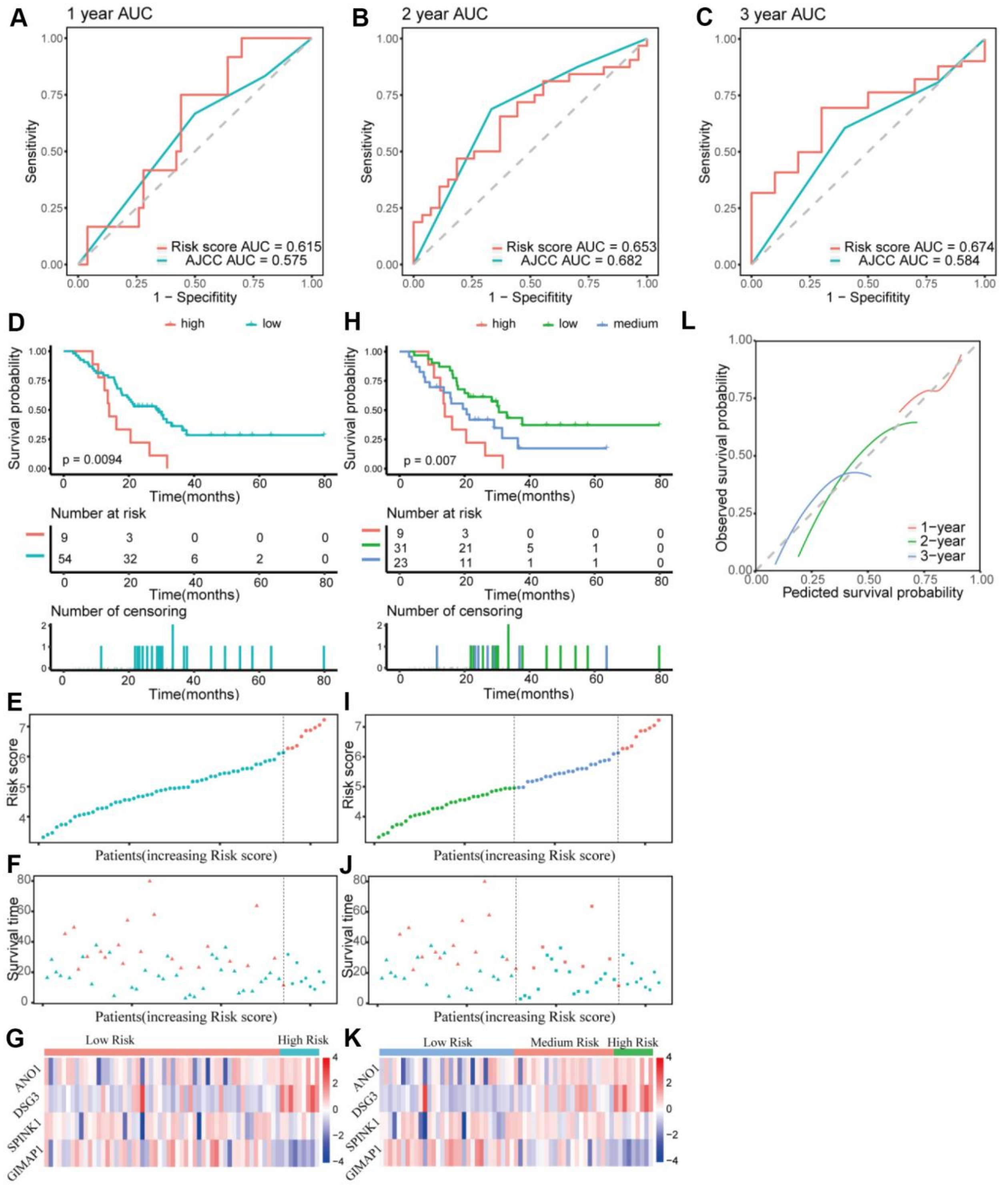


**Supplementary Figure 2. External validation of the prognostic gene signature.** (**A**–**C**) Time dependent ROC curves for 1-, 2- and 3-years overall survival predictions of genes signature in GSE57495, and AJCC stage is the control. (**D**–**G**) Kaplan-Meier survival curves of the four-gene signature and distribution of patient survival and risk score in different groups when patients in GSE57495 were divided into two groups. (**D**) Kaplan-Meier survival curves. (**E**) Distribution of the risk score. (**F**) Distribution of the survival time. Circle shape stands for high-risk group while triangle shape for low-risk group. Red stands for survival and green stands for dead. (**G**) Heatmap of the expression of the four genes. (**H**–**K**) Kaplan-Meier survival curves of the four-gene signature and distribution of patient survival and risk score in different groups when patients in GSE57495 were divided into three groups. (**H**) Kaplan-Meier survival curves. (**I**) Distribution of the risk score. (**J**) Distribution of the survival time. (**K**) Heatmap of the expression of the four genes. (**L**) Calibration plot for validation of the gene signature in GSE57495.

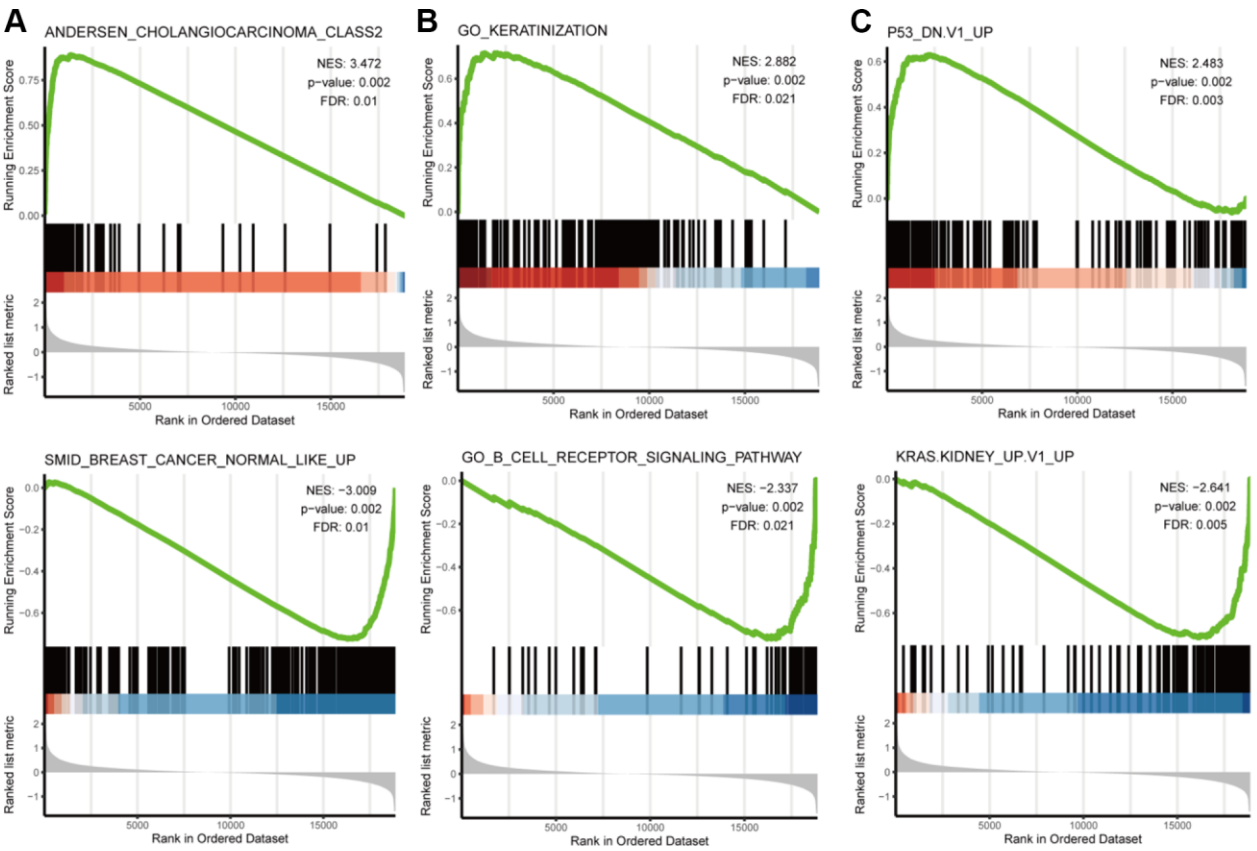


**Supplementary Figure 3. GSEA for other MSigDB gene sets.** Upregulated and downregulated enriched pathways with top NES were showed. (**A**) C2 (curated gene sets). (**B**) C5 (GO gene sets). (**C**) C6 (oncogenic gene sets).

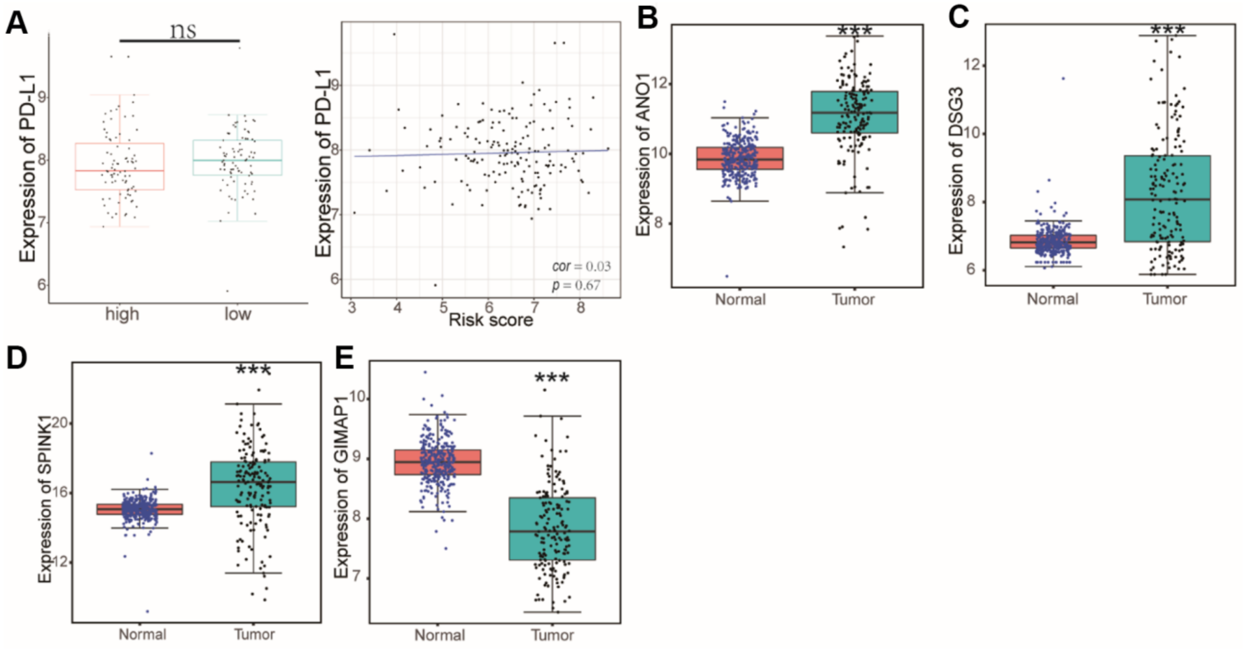


**Supplementary Figure 4. Expression of PD-L1 and four genes in signature.** (**A**) Correlation between expression of PD-L1 and risk score. (**B**–**E**) The mRNA expression of four genes in signature.
