# Supplementary material for: A novel gene signature for prognosis prediction and chemotherapy response in patients with pancreatic cancer
Source: Aging (Albany NY). 2021 Apr 26;13(9):12493–513. doi: 10.18632/aging.202922 (PMC8148498; doi:10.18632/aging.202922)
Supplement: Supplementary Table 1 [file aging-13-202922-s002.doc]

**Supplementary Table 1. Clinical characteristics of pancreatic cancer patients in the TCGA dataset**.

| Clinical characteristic | All (n = 165) | Train set (n = 115) | Test set (n = 50) |
| --- | --- | --- | --- |
| Overall survival time(day) | 591.12 ± 478.99 | 609.38 ± 495.23 | 549.14 ± 441.28 |
| Age at diagnosis | 64.53 ± 10.84 | 64.25 ± 10.88 | 65.18 ± 10.83 |
| Tumor dimension(cm) | 3.91 ± 1.70 | 3.77 ± 1.59 | 4.20 ± 1.89 |
| Survival status | | | |
| Alive | 76 (46.06%) | 58(50.43%) | 18(36%) |
| Dead | 89(53.94%) | 57(49.57%) | 32(64%) |
| Gender | | | |
| Male | 90(54.55%) | 61(53.04%) | 29(58%) |
| Female | 75(45.45%) | 54(46.96%) | 21(42%) |
| AJCC 7^th^ Stage | | | |
| I | 1(0.61%) | 0(0.00%) | 1(2.00%) |
| IA | 4(2.42%) | 4(3.48%) | 0(0.00%) |
| IB | 13(7.88%) | 13(11.3%) | 0(0.00%) |
| IIA | 26(15.76%) | 17(14.78%) | 9(18%) |
| IIB | 112(67.88%) | 75(65.22%) | 37(74%) |
| III | 3(1.82%) | 2(1.74%) | 1(2%) |
| IV | 4(2.42%) | 2(1.74%) | 2(4%) |
| Not Available | 2(1.21%) | 2(1.74%) | 0(0.00%) |
| T | | | |
| T1 | 6(3.64%) | 5(4.35%) | 1(2.00%) |
| T2 | 20(12.12%) | 18(15.65%) | 2(4%) |
| T3 | 134(81.21%) | 88(76.52%) | 46(92%) |
| T4 | 3(1.82%) | 2(1.74%) | 1(2%) |
| Tx | 2(1.21%) | 2(1.74%) | 0(0.00%) |
| N | | | |
| N0 | 45(27.27%) | 34(29.57%) | 11(22%) |
| N1 | 112(67.88%) | 75(65.22%) | 37(74%) |
| N1b | 4(2.42%) | 3(2.61%) | 1(2%) |
| Nx | 4(2.42%) | 3(2.61%) | 1(2.00%) |
| M | | | |
| M0 | 74(44.85%) | 53(46.09%) | 21(42%) |
| M1 | 4(2.42%) | 2(1.74%) | 2(4%) |
| Mx | 87(52.73%) | 60(52.17%) | 27(54%) |
| Sites | | | |
| Body of Pancreas | 14(8.48%) | 12(10.43%) | 2(4%) |
| Head of Pancreas | 128(77.58%) | 86(74.78%) | 42(84%) |
| Tail of Pancreas | 12(7.27%) | 10(8.70%) | 2(4.00%) |
| Other | 11(6.67%) | 7(6.09%) | 4(8.00%) |
| Pathology | | | |
| Pancreas-Adenocarcinoma Ductal Type | 140(84.85%) | 97(84.35%) | 43(86.00%) |
| Pancreas-Adenocarcinoma-Other Subtype | 25(15.15%) | 18(15.65%) | 7(14.00%) |
| Grade | | | |
| G1 | 27(16.36%) | 21(18.26%) | 6(12.00%) |
| G2 | 89(53.94%) | 64(55.65%) | 25(50.00%) |
| G3 | 47(28.48%) | 28(24.35%) | 19(38.00%) |
| G4 | 1(0.61%) | 1(0.87%) | 0(0.00%) |
| Gx | 1(0.61%) | 1(0.87%) | 0(0.00%) |
| Neoadjuvant | | | |
| No | 164(99.39%) | 115(100.00%) | 49(98.00%) |
| Yes | 1(0.61%) | 0(0.00%) | 1(2.00%) |
| Chemotherapy | | | |
| No | 51(30.91%) | 34(29.57%) | 17(34.00%) |
| Yes | 114(69.09%) | 81(70.43%) | 33(66.00%) |
| Radiation | | | |
| No | 109(66.06%) | 75(65.22%) | 34(68.00%) |
| Yes | 42(25.45%) | 31(26.96%) | 11(22.00%) |
| [Unknown] | 14(8.48%) | 9(7.83%) | 5(10.00%) |
| Molecular therapy | | | |
| No | 42(25.45%) | 29(25.22%) | 13(26.00%) |
| Yes | 86(52.12%) | 63(54.78%) | 23(46.00%) |
| [Unknown] | 37(22.42%) | 23(20.00%) | 14(28.00%) |
| Surgery | | | |
| Whipple | 127(76.97%) | 83(72.17%) | 44(88.00%) |
| Total Pancreatectomy | 2(1.21%) | 1(0.87%) | 1(2.00%) |
| Distal Pancreatectomy | 22(13.33%) | 18(15.65%) | 4(8.00%) |
| Other Method | 12(7.27%) | 11(9.57%) | 1(2.00%) |
| [Not Available] | 2(1.21%) | 2(1.74%) | 0(0.00%) |
| Residual tumor | | | |
| R0 | 96(58.18%) | 67(58.26%) | 29(58.00%) |
| R1 | 51(30.91%) | 33(28.7%) | 18(36.00%) |
| R2 | 5(3.03%) | 4(3.48%) | 1(2.00%) |
| Rx | 13(7.88%) | 11(9.57%) | 2(4.00%) |
| Smoking | | | |
| Lifelong Non-smoker | 60(36.36%) | 46(40%) | 14(28.00%) |
| Current smoker | 18(10.91%) | 13(11.3%) | 5(10.00%) |
| Current reformed smoker for < or = 15 years | 22(13.33%) | 18(15.65%) | 4(8.00%) |
| Current reformed smoker for > 15 years | 27(16.36%) | 17(14.78%) | 10(20.00%) |
| Current Reformed Smoker, Duration Not Specified | 7(4.24%) | 6(5.22%) | 1(2.00%) |
| [Unknown] | 31(18.79%) | 15(13.04%) | 16(32.00%) |
| Drinking | | | |
| No | 61(36.97%) | 39(33.91%) | 22(44.00%) |
| Yes | 92(55.76%) | 69(60.00%) | 23(46.00%) |
| [Not Available] | 12(7.27%) | 7(6.09%) | 5(10.00%) |
| History of pancreatitis | | | |
| No | 119(72.12%) | 86(74.78%) | 33(66.00%) |
| Yes | 13(7.88%) | 9(7.83%) | 4(8.00%) |
| [Not Available] | 33(20.00%) | 20(17.39%) | 13(26.00%) |
| History of diabetes | | | |
| No | 102(61.82%) | 76(66.09%) | 26(52.00%) |
| Yes | 34(20.61%) | 23(20.00%) | 11(22.00%) |
| [Not Available] | 29(17.58%) | 16(13.91%) | 13(26.00%) |
| Family history of cancer | | | |
| No | 42(25.45%) | 28(24.35%) | 14(28.00%) |
| Yes | 60(36.36%) | 48(41.74%) | 12(24.00%) |
| [Not Available] | 63(38.18%) | 39(33.91%) | 24(48.00%) |
| Response after chemotherapy (n = 71) | | | |
| Clinical Progressive Disease | 35(49.30%) | 23(46.94%) | 12(54.55%) |
| Stable Disease | 3(4.23%) | 2(4.08%) | 1(4.55%) |
| Partial Response | 4(5.63%) | 3(6.12%) | 1(4.55%) |
| Complete Response | 29(40.85%) | 21(42.86%) | 8(36.36%) |
